# Diagnostic Performance of Transabdominal Ultrasonography for Pancreatic Fat in Adults with Metabolic Risk Factors for NAFPD: Comparison with a Study-Defined Two-Echo MRI Index

**DOI:** 10.3390/diagnostics16182959

**Published:** 2026-09-13

**Authors:** Anel Zhangereyeva, Venera Rakhmetova, Nurlan Baisynov, Aitore Zhangereyev

**Affiliations:** 1 Doctoral Program, Non-Profit Joint-Stock Company “Astana Medical University”, 49A Beibitshilik Street, Astana 010000, Kazakhstan; 2 Department of Internal Diseases with Courses in Nephrology, Hematology, Allergology, and Immunology, Non-Profit Joint-Stock Company “Astana Medical University”, 49A Beibitshilik Street, Astana 010000, Kazakhstan; venerarakhmetova@gmail.com; 3 Research Department, Non-Governmental Educational Institution “Kazakhstan-Russian Medical University”, 51/53 Abylai Khan Avenue, Almaty 050004, Kazakhstan; 4 Center for Analytical Testing LLP, 5 Kaiym Mukhamedkhanov Street, Astana 010000, Kazakhstan; testowallker@gmail.com

**Keywords:** pancreas, pancreatic fat, fatty pancreas, ultrasonography, magnetic resonance imaging, diagnostic accuracy, technical visualization, STARD

## Abstract

**Background/Objectives:** To evaluate an exploratory ordinal transabdominal ultrasonography score against a study-defined semiquantitative two-echo chemical-shift MRI pancreatic fat index ≥ 5%. **Methods:** This prospective paired diagnostic accuracy study included 150 adults aged 30–60 years with metabolic abnormalities or risk factors and technically adequate ultrasonography. A score ≥ 1 was the primary positive index-test result. Diagnostic accuracy, ROC AUC, and ordinal correspondence were evaluated. **Results:** After nine clinical exclusions, pancreatic ultrasonography was technically evaluable in 150 of 170 participants (88.2%; 95% CI, 82.5–92.3%). For the study-defined MRI index ≥ 5%, sensitivity was 76.8% (95% CI, 66.6–84.6%), specificity was 77.9% (95% CI, 66.7–86.2%), accuracy was 77.3% (95% CI, 70.0–83.3%), and AUC was 0.812 (95% CI, 0.748–0.876). Exact four-category agreement was 45.3%, and quadratically weighted kappa was 0.504 (95% CI, 0.387–0.612). **Conclusions:** These estimates describe agreement and discrimination relative to the study-defined two-echo MRI index in selected adults with metabolic risk and technically adequate ultrasonography. They should not be interpreted as diagnostic accuracy for histologically confirmed pancreatic steatosis or standardized MRI-PDFF-defined disease. The exploratory score is not a quantitative substitute for MRI and requires reproducibility assessment and external validation.

## 1. Introduction

Pancreatic fat accumulation has historically been described using several terms, including pancreatic steatosis, pancreatic lipomatosis, fatty pancreas, intrapancreatic fat deposition, and nonalcoholic fatty pancreas disease (NAFPD) [1,2,3]. A 2026 international multidisciplinary consensus recommended ‘fatty pancreas’ as the broad standardized term regardless of etiology [1]. In the present study, NAFPD is retained only as a contextual term for the metabolically at-risk population and when describing earlier literature. Its retention in the title identifies the clinical research context in which metabolic risk and exclusion of clinically relevant alcohol exposure and major secondary pancreatic causes are considered; it does not define the reference diagnosis. The study endpoint itself is a study-defined MRI-derived pancreatic fat index and is not presented as a validated disease definition. A recent comprehensive review similarly emphasizes that the absence of harmonized diagnostic criteria remains a major barrier to comparing imaging studies and translating fatty-pancreas findings into clinical practice [4,5].

Interest in pancreatic fat accumulation has increased alongside the global burden of obesity, insulin resistance, and other components of metabolic syndrome [2,3,6,7]. Reported prevalence varies substantially across studies because of differences in source populations, imaging modalities, image interpretation, and diagnostic thresholds [6,7,8,9,10,11]. Population-based and clinical studies have associated pancreatic fat with increasing age, general and abdominal adiposity, type 2 diabetes mellitus, arterial hypertension, dyslipidemia, metabolic syndrome, and fatty liver disease [7,8,9,10,11]. However, these associations are not uniformly independent after adjustment for confounding factors, and the causal and prognostic significance of pancreatic fat remains uncertain [3,12]. An updated meta-analysis of 18 studies, including more than 111,000 individuals, estimated an overall fatty-pancreas prevalence of about 21%, with substantial variation according to imaging modality, further illustrating the importance of method-specific definitions [13]. More recent MRI studies also support close associations of pancreatic fat with obesity and visceral adiposity [14].

Potential clinical consequences have been linked to impaired glucose metabolism, reduced beta-cell functional reserve, local inflammatory signaling, and altered exocrine pancreatic function [3,8,12,15]. Evidence relating pancreatic fat to insulin secretion and beta-cell function is inconsistent. Some studies have reported inverse associations, whereas others found substantial attenuation after accounting for age, visceral adiposity, and systemic insulin resistance [3,8,15]. Pancreatic fat may therefore represent an imaging phenotype of metabolic dysfunction, but its independent role as a disease entity and prognostic marker requires further clarification. Longitudinal evidence has linked MRI-defined fatty pancreas with subsequent development of diabetes, while reviews of exocrine outcomes show that the clinical significance remains heterogeneous and incompletely resolved [16,17,18].

Histological confirmation is not routinely feasible because pancreatic biopsy is invasive, the organ is retroperitoneal, and heterogeneous fat distribution creates a risk of sampling error. Diagnostic assessment therefore relies mainly on imaging [1,3,12]. Transabdominal ultrasonography is inexpensive, widely available, and free of ionizing radiation. Findings suggestive of pancreatic fat accumulation include increased parenchymal echogenicity relative to the liver or renal cortex, heterogeneous echotexture, posterior acoustic attenuation, and reduced visualization of pancreatic margins, vascular landmarks, and the pancreatic duct [10,11,19,20,21,22,23].

Ultrasonographic assessment nevertheless has important limitations. Echogenicity is influenced not only by fat but also by fibrosis, age, equipment, gain settings, transducer frequency, and operator technique. Image quality may be impaired by obesity, pancreatic depth, and gastric or intestinal gas [20,21,22,23]. Comparison with the liver may be confounded by coexisting hepatic steatosis, while comparison with the kidney is not always reproducible because of differences in depth and acoustic windows. Published ultrasonography classifications differ in the number of grades and the signs included, and no universally validated transabdominal ultrasonography scale for quantitative pancreatic fat assessment is currently available [1,20,21,22,23]. Recent studies further show an association between ultrasound-detected pancreatic steatosis and metabolic syndrome, while contemporary ultrasound reviews emphasize that pancreatic appearance and measurements are influenced by age, body habitus, acoustic windows, and examination technique, supporting standardized acquisition and interpretation [24,25].

MRI can separate water and fat signals without ionizing radiation. Multi-echo Dixon-based techniques can generate proton density fat fraction maps while correcting major physical sources of bias, including T1 weighting, T2* decay, and the multi-peak spectrum of fat [26,27,28,29]. MRI-derived pancreatic fat measurements have been compared with histological findings in surgical cohorts and have also been used in population studies of prevalence and clinical associations [27,28]. However, pancreatic MRI remains technically challenging because the gland is small, irregularly shaped, and surrounded by peripancreatic fat. Results depend on sequence design, correction methods, region-of-interest placement, slice selection, and averaging strategy [26,27,28,29]. Unlike the liver, the pancreas does not yet have universally accepted thresholds or validated severity categories [1,3,29]. Methodological studies using whole-pancreas or harmonized MRI protocols have shown that reproducibility can be improved by standardized acquisition and sampling, whereas accurate measurement of low-level fat fractions remains sensitive to reconstruction algorithms and correction of physical confounders [30,31,32]. A 2026 systematic review and meta-analysis found strong correlations between chemical-shift-encoded MRI fat fractions and histologic measurements, while also calling for optimized and universally applicable protocols [33].

These limitations create a clinically relevant gap. Transabdominal ultrasonography is suitable for first-line assessment, but its ability to identify elevated pancreatic fat and to reflect increasing fat burden must be tested against a more objective imaging measure. Previous studies have often used binary definitions of a hyperechoic pancreas, endoscopic ultrasonography, computed tomography, or heterogeneous MRI techniques, limiting direct comparison across studies [6,7,20,21,22,23]. A recent study of 46 participants compared an improved ultrasonography approach with MRI mDIXON-Quant using a pancreatic fat fraction threshold > 6.2% and reported stronger agreement than a traditional ultrasonography approach; however, its small cohort and different imaging protocol limit direct comparison [34]. Evidence remains limited regarding the diagnostic performance of individual thresholds of an ordinal ultrasonography scale, its overall discrimination, and its correspondence with study-defined MRI categories.

The primary objective was to evaluate the diagnostic performance of an exploratory ordinal ultrasonography score ranging from 0 to 3 for detecting a study-defined semiquantitative two-echo MRI-derived pancreatic fat index ≥ 5%. Secondary objectives were to estimate sensitivity and specificity at different ultrasonography thresholds, quantify discrimination using ROC analysis, examine associations with the continuous MRI-derived index, and assess ordinal correspondence between ultrasonography and operational MRI categories. The study did not evaluate diagnostic accuracy against histology or standardized MRI-PDFF and did not seek to validate a clinical definition of fatty pancreas.

## 2. Materials and Methods

### 2.1. Study Design and Setting

A prospective observational diagnostic accuracy study with a paired imaging design was conducted in Astana, Kazakhstan. Recruitment, clinical and anthropometric assessment, and transabdominal pancreatic ultrasonography were performed at Life Medicus Multidisciplinary Medical Center. MRI was performed at Lider MRI Center. Both institutions examined the same study cohort; the design should therefore be interpreted as a single-cohort paired study rather than an independent multicenter validation.

The study was conducted from 25 October 2025 to 30 April 2026. Clinical data and imaging examinations were collected prospectively. Transabdominal ultrasonography was performed first, followed by MRI. Standardization of the original ultrasonography reports and stored images into an ordinal score of 0–3, together with the final diagnostic accuracy analysis, was completed after the analytical database had been finalized.

Potential participants were identified among patients presenting to the research center, individuals referred by physicians, participants in preventive examinations, and specifically invited individuals who met the study criteria. This mixed recruitment strategy did not constitute a strictly consecutive or random series.

After eligibility assessment and written informed consent, participants underwent clinical and anthropometric assessment, transabdominal ultrasonography, and MRI. The interval between the index test and reference measure did not exceed one day. Transabdominal ultrasonography was treated as the index test, and a calculated semiquantitative MRI-derived pancreatic fat index was treated as the reference measure. Reporting followed the STARD 2015 recommendations for diagnostic accuracy studies. The completed STARD 2015 checklist is provided as Checklist S1 in the Supplementary Materials.

### 2.2. Participants and Eligibility Criteria

Potential participants were men and women aged 30–60 years with metabolic abnormalities or metabolic risk factors. Metabolic risk was defined by one or more of the following: overweight or obesity, abnormal glucose metabolism, dyslipidemia, or arterial hypertension.

Inclusion criteria were:

- Age 30–60 years;
- Metabolic abnormalities or metabolic risk factors;
- Available clinical, medical-history, and anthropometric data;
- Completed transabdominal pancreatic ultrasonography;
- Completed MRI suitable for calculating the MRI-derived pancreatic fat index;
- Available information on alcohol consumption;
- Written informed consent for participation and use of de-identified results in scientific publications.

Exclusion criteria were:

- Acute or chronic pancreatitis;
- Alcohol-associated pancreatitis;
- Alcohol dependence or alcohol-associated liver disease;
- Viral or autoimmune hepatitis;
- Severe hepatic failure;
- Cystic fibrosis;
- Hemochromatosis;
- Previous pancreatic surgery;
- Use of medications capable of causing fatty replacement of the pancreas;
- Active or suspected malignant disease;
- Another severe comorbidity likely to substantially affect the pancreas or the metabolic variables under study;
- Claustrophobia or another contraindication to MRI;
- Technically inadequate pancreatic visualization;
- Absence of an evaluable result from either imaging method;
- Age younger than 30 years or older than 60 years.

Alcohol exposure was assessed using a clinical history interview, participant self-report, and available medical records. Men reporting more than 80 g of pure ethanol per day and women reporting more than 40 g per day were excluded. Individuals with established alcohol dependence, alcohol-associated liver disease, or alcohol-associated pancreatitis were also excluded. A validated quantitative alcohol questionnaire was not used; therefore, residual misclassification of alcohol exposure cannot be fully excluded.

**Figure 1:**
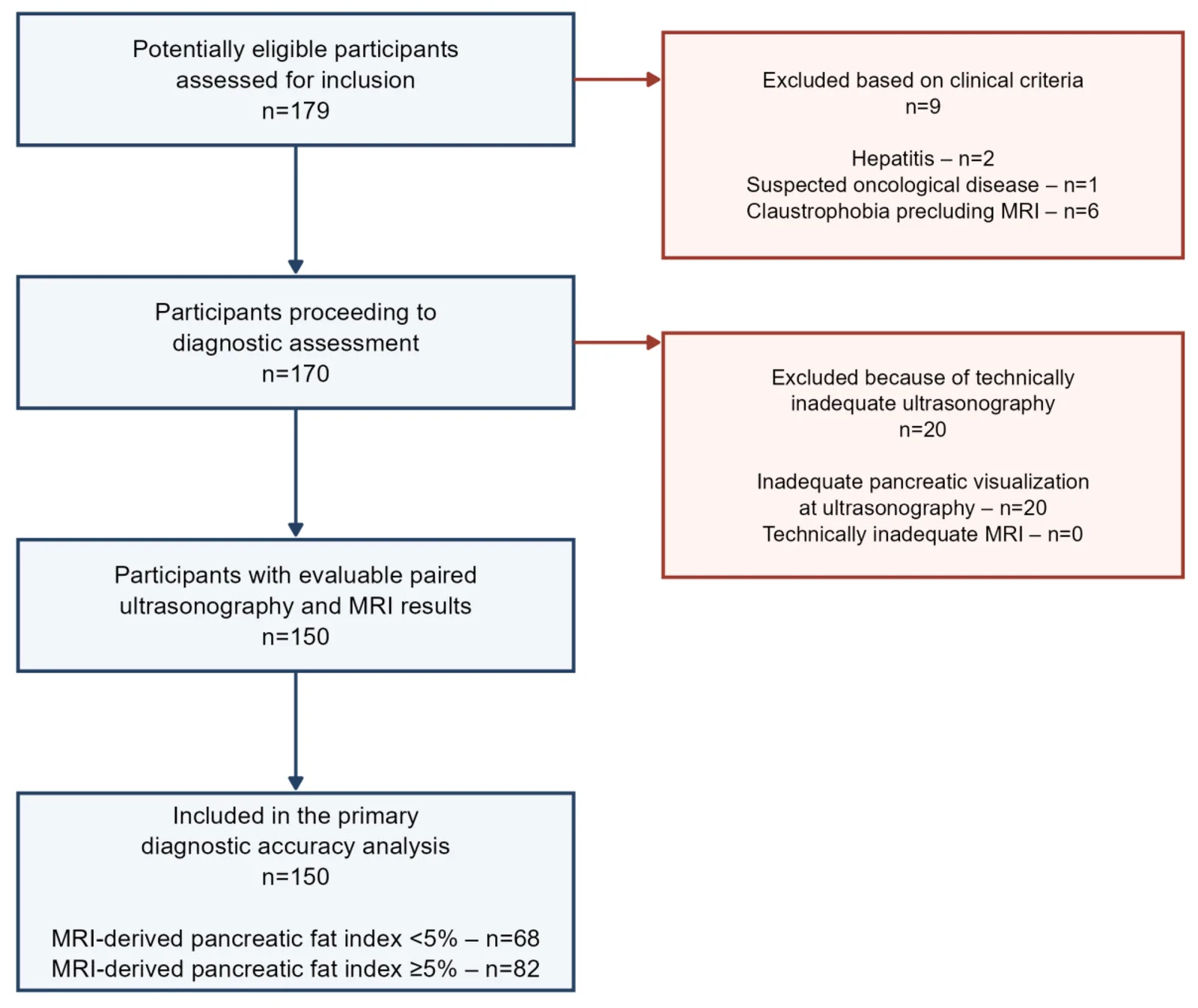
STARD 2015 flow diagram of participant selection and inclusion in the diagnostic accuracy analysis. MRI, magnetic resonance imaging. Blue boxes indicate participant flow through the study, whereas red boxes indicate exclusions.

A total of 179 potentially eligible individuals were assessed. Nine were excluded on clinical grounds: hepatitis (*n* = 2), suspected malignant disease (*n* = 1), and claustrophobia precluding MRI (*n* = 6). Of the 170 participants remaining after these exclusions, 20 had technically inadequate pancreatic visualization on transabdominal ultrasonography. The technical visualization yield was therefore 150/170 (88.2%; 95% CI, 82.5–92.3%), and the technical non-visualization rate was 20/170 (11.8%; 95% CI, 7.7–17.5%). Individual anthropometric variables and MRI reference results for these 20 participants were not available in the finalized analytical dataset used for this revision; consequently, an included-versus-excluded comparison and exact full-cohort accuracy analysis could not be performed. No technically inadequate or indeterminate MRI results occurred. The final paired diagnostic accuracy analysis included 150 participants (Figure 1).

### 2.3. Clinical and Anthropometric Assessment

Clinical information was recorded using a standardized case report form. Variables included age, sex, smoking status, arterial hypertension, diabetes mellitus, current medications, and relevant medical history.

Body weight and height were measured during the clinical examination. Body mass index (BMI) was calculated as body weight in kilograms divided by height in meters squared: BMI = weight (kg)/height^2^ (m^2^). BMI-defined obesity was defined as BMI of at least 30 kg/m^2^.

Waist circumference was measured with the participant standing, feet shoulder-width apart, and arms relaxed. The tape was positioned midway between the lower margin of the last palpable rib and the top of the iliac crest. Three measurements were obtained at the end of a normal expiration, and their arithmetic mean was used in the analysis.

Arterial hypertension was defined by a previous diagnosis, current antihypertensive treatment, or blood pressure measurements obtained during the clinical examination. Diabetes mellitus was defined by a previous diagnosis, glucose-lowering treatment, or laboratory findings. Current smoking was defined as use of tobacco products during the 30 days preceding enrollment.

### 2.4. Index Test: Transabdominal Ultrasonography

Transabdominal pancreatic ultrasonography was performed at Life Medicus Multidisciplinary Medical Center using a Samsung S40 ultrasound system manufactured in 2015 (Samsung Medison Co., Ltd., Seoul, Republic of Korea) with a 2–7.5 MHz convex transducer.

Examinations were performed after 6–12 h of fasting. No additional preparation was used. Scanning was conducted with participants in supine and lateral positions using transverse, longitudinal, and oblique planes. The pancreatic head, body, and tail were assessed whenever technically possible.

The original examinations were performed and interpreted by one senior ultrasonography physician with 15 years of experience. MRI results were unavailable because MRI was performed after ultrasonography. Standard clinical information was available to the ultrasonography physician.

The original ultrasonography reports recorded pancreatic echogenicity, its relationship to hepatic echogenicity, echotexture homogeneity, visibility of pancreatic margins, visualization of the head, body, and tail, and the presence of acoustic attenuation.

For the present diagnostic analysis, information from the original reports and stored images was standardized using an exploratory ordinal scale from 0 to 3:

0 points: no diffuse increase in echogenicity; homogeneous echotexture; preserved visualization of the pancreatic margins, head, body, and tail; and no substantial acoustic attenuation.
1 point: mild diffuse increase in echogenicity relative to the liver and/or minimal diffuse changes, with preserved visualization of pancreatic margins and major anatomical regions.
2 points: moderate diffuse increase in echogenicity accompanied by heterogeneous echotexture; partial reduction in visualization of pancreatic margins or individual regions due to acoustic attenuation was permitted.
3 points: marked diffuse increase in echogenicity accompanied by at least one additional feature: pronounced diffuse or heterogeneous changes, substantial reduction in visualization of pancreatic margins and regions due to acoustic attenuation, or ultrasonographic features interpreted as lipomatosis.

The phrase “ultrasonographic features of lipomatosis” was not accepted as the sole basis for assigning a score of 3 because it represents an overall diagnostic interpretation rather than a single reproducible imaging sign.

The original descriptions and stored images were reviewed jointly by the ultrasonography physician and a second-year doctoral researcher who was also a gastroenterologist, and a consensus score was assigned. MRI results were unavailable during this process. Independent initial scores were not recorded, and independent rescoring or repeat scoring was not performed; therefore, inter-reader and intra-reader reproducibility could not be estimated. The retrospectively standardized 0–3 score is exploratory and has not been validated for reproducibility.

The primary index-test result was the original binary ultrasonography interpretation of the presence or absence of diffuse pancreatic changes. During subsequent standardization, a negative original result corresponded to a score of 0 and a positive result corresponded to a score of at least 1. Accordingly, a score of at least 1 was used as the primary positive threshold. The 0–3 scale and thresholds of at least 2 and at least 3 were treated as secondary exploratory measures.

When the acoustic window was inadequate and evaluable pancreatic visualization could not be obtained, the ultrasonography result was classified as technically inadequate rather than negative. Twenty of 170 participants remaining after clinical exclusions met this definition. Among the 150 participants with technically adequate examinations, the ultrasonography score was available for all participants, with no missing or indeterminate index-test results.

### 2.5. Reference Measure: Calculated MRI-Derived Pancreatic Fat Index

MRI was performed at Lider MRI Center using a 1.5-T Philips Achieva system (Philips Medical Systems Nederland B.V., Best, The Netherlands). The same scanner, sequence, and calculation procedure were used for all participants.

Two-echo chemical-shift MRI was selected as the study reference measure because it provided a uniform, non-invasive semiquantitative comparator for all participants with evaluable paired examinations, was acquired within one day of ultrasonography using the same scanner and protocol, and allowed regional sampling of the pancreatic head, body, and tail. Histological verification was not performed because pancreatic biopsy had no clinical indication and invasive sampling solely for research was not justified. This choice improved internal consistency relative to subjective echogenicity assessment but did not establish the MRI-derived index as a validated gold standard or as standardized MRI-PDFF.

The protocol included a two-echo gradient-echo chemical-shift dual FFE breath-hold sequence with opposed-phase and in-phase images. mDIXON, IDEAL-IQ, magnetic resonance spectroscopy, and multi-echo quantitative MRI-PDFF were not used.

The sequence parameters were:

- First echo time (TE1), 2.3 ms;
- Second echo time (TE2), 4.6 ms;
- Repetition time, the shortest value automatically selected by the system;
- Flip angle, 80°;
- Slice thickness, 7 mm;
- Interslice gap, 1 mm;
- Matrix, 256 × 152;
- Field of view, 405 × 321 × 199 mm;
- Voxel size, 1.41 × 1.83 × 7 mm;
- Number of echoes, 2;
- SENSE parallel imaging factor, 1.8;
- Acquisition during an inspiratory breath-hold.

The MRI-derived pancreatic fat index was calculated manually from the signal intensities on in-phase and opposed-phase images using the following expression:

*MRI-derived pancreatic fat index* (%) = [(*SIin-phase* − *SIopposed-phase*)/(2 × *SIin-phase*)] × 100

where *SIin-phase* is the in-phase signal intensity and *SIopposed-phase* is the opposed-phase signal intensity. Calculations were performed manually in Microsoft Excel. The final value was the arithmetic mean of all technically suitable measurements.

Regions of interest (ROIs) were placed manually in the pancreatic head, body, and tail. One or two ROIs were placed in each anatomical region, resulting in three to six ROIs per participant. Circular or oval ROIs were individually sized and placed on one or more consecutive slices.

Large vessels, the main pancreatic duct, focal lesions, regions with marked artifacts, and peripancreatic fat were excluded from ROIs. The final MRI-derived pancreatic fat index was calculated as the arithmetic mean of all suitable ROIs.

No correction was applied for T1 weighting, T2* decay, noise bias, the multi-peak fat spectrum, or B0 and B1 field inhomogeneity. These omissions may introduce systematic and spatially variable measurement error because water and fat signals can be influenced by sequence weighting, echo-time-dependent signal loss, spectral complexity, field non-uniformity, and low-signal noise. The magnitude and direction of the resulting bias cannot be determined from the present data. Misclassification is especially plausible near the 5% threshold and may either attenuate or inflate the observed sensitivity, specificity, and AUC. The resulting measure was therefore treated as a calculated semiquantitative two-echo chemical-shift MRI index rather than standardized MRI-PDFF.

Initial ROI placement and signal-intensity measurements were performed by three radiologists with more than two years of MRI experience. All selected slices, ROI locations, signal-intensity measurements, and calculations were subsequently reviewed by a senior radiologist with more than eight years of MRI experience, who made the final decision regarding measurement suitability and the final index. MRI readers were not provided with ultrasonography results. Because independent duplicate ROI measurements of the same examinations were not retained, this review process did not permit estimation of inter-reader or intra-reader MRI measurement reproducibility.

The primary reference outcome was a study-defined MRI-derived pancreatic fat index ≥ 5%. No validation evidence was identified supporting 5% as a diagnostic cutoff specifically for the two-echo signal-intensity index used in this study. The 5% value was therefore retained solely as a pragmatic, study-defined operational lower boundary for the primary binary endpoint and the exploratory categories; it was not selected by optimizing the ROC curve in this dataset. Published MRI studies have reported low single-digit pancreatic fat values, but acquisition and analysis methods vary, and no universally accepted normal threshold exists [1,3,28,29]. To evaluate threshold dependence, we additionally analyzed the literature-informed value > 6.2% reported with a different MRI method [34]. Four categories were used only for exploratory ordinal analysis:

− <5%;
− 5 to <10%;
− 10 to <20%;
− ≥20%.

These intervals were operational categories specific to the present study. They were not interpreted as validated MRI-PDFF grades, established clinical stages, or a disease definition of fatty pancreas. Throughout the manuscript, ‘MRI-reported pancreatic fat’ refers only to this study-defined semiquantitative two-echo MRI-derived index.

### 2.6. Interval Between Ultrasonography and MRI

Transabdominal ultrasonography was performed before MRI, and the interval between the index test and reference measure did not exceed one day. Ultrasonography was completed at Life Medicus Multidisciplinary Medical Center, after which the participant was referred to Lider MRI Center.

Between the examinations, participants did not receive treatment, infusion therapy, food, or another intervention expected to affect pancreatic imaging findings or the calculated MRI-derived pancreatic fat index.

All 150 participants in the final diagnostic analysis underwent both examinations regardless of the result of the first test. The same MRI protocol and calculation procedure were applied to all participants. No technically inadequate or indeterminate MRI results occurred.

The final analytical database contained no missing ultrasonography scores or MRI-derived pancreatic fat index values. Missing-value imputation was not performed. No adverse events related to ultrasonography or MRI were observed.

### 2.7. Diagnostic Outcomes

The primary objective was to evaluate the diagnostic performance of the ordinal ultrasonography score for detecting the study-defined MRI-derived pancreatic fat index of at least 5%.

The primary positive ultrasonography result was a score of at least 1. Sensitivity, specificity, positive and negative predictive values, overall accuracy, positive and negative likelihood ratios, and diagnostic odds ratios were calculated for this threshold.

Thresholds of at least 2 and at least 3 were evaluated as secondary exploratory ultrasonography thresholds.

Additional analyses included:

- Receiver operating characteristic analysis of the ordinal ultrasonography score;
- Association between the ultrasonography score and the continuous MRI-derived pancreatic fat index;
- Cross-classification of the four ultrasonography score categories and four study-defined MRI categories;
- Exact agreement and agreement within one category;
- Linear- and quadratic-weighted Cohen’s kappa;
- Sensitivity analyses using MRI thresholds of >6.2%, ≥10%, and ≥20%.

The threshold of >6.2% was treated as a method-dependent, literature-informed exploratory threshold [6,34] and was not used to construct the primary four MRI categories. The threshold of at least 10% was secondary, while findings for at least 20% were descriptive because only nine participants met this criterion.

### 2.8. Sample Size and Data Completeness

The final analytical sample included all eligible participants with evaluable paired ultrasonography and MRI results during the study period, yielding 150 participants.

During planning of the broader study, sample size was considered in relation to a proposed multivariable logistic regression model, using at least 10 outcome events per estimated parameter as a practical rule. However, the retained study documentation did not contain a reproducible calculation specifying the number of parameters, expected outcome prevalence, and allowance for technically inadequate examinations. This approach was therefore not treated as a documented a priori sample-size calculation for the present diagnostic accuracy analysis.

No prospective sample-size calculation was performed specifically for this diagnostic accuracy study. The sample size was determined by the number of participants enrolled during the study period who had complete index-test and reference-measure results. Precision was described using 95% confidence intervals. For the primary threshold, the 95% confidence intervals were 66.6–84.6% for sensitivity and 66.7–86.2% for specificity.

A retrospective Buderer calculation based on the observed prevalence, sensitivity, and specificity indicated that approximately 146 participants would be required for an absolute precision of about 10 percentage points at the 95% confidence level. This was a descriptive assessment of achieved precision and was not interpreted as an a priori power calculation.

The achieved sample size did not provide similar precision across all four MRI-derived pancreatic fat categories, particularly the category of at least 20%, which included nine participants. Multicategory analyses and analyses using the at least 20% threshold were therefore treated as exploratory and descriptive.

No data were missing for age, BMI, waist circumference, sex, arterial hypertension, diabetes mellitus, smoking, obesity, ultrasonography score, or MRI-derived pancreatic fat index. Statistical imputation was not performed.

### 2.9. Statistical Analysis

Statistical analysis was performed in R version 4.5.2 (R Foundation for Statistical Computing, Vienna, Austria) under Windows 11. The principal packages were pROC version 1.19.0.1, dplyr version 1.2.0, tidyr version 1.3.2, tibble version 3.3.0, purrr version 1.1.0, stringr version 1.6.0, ggplot2 version 4.0.3, readxl version 1.4.5, and openxlsx version 4.2.8.1.

The analytical database was finalized before the definitive analysis and contained 150 unique participants with complete paired ultrasonography and MRI results.

For baseline comparisons, distributions of continuous variables were assessed separately in the MRI-derived pancreatic fat index groups below 5% and at least 5% using the Shapiro–Wilk test. Variables that were normally distributed in both groups were summarized as mean and standard deviation and compared using Welch’s *t* test; effect size was expressed as Hedges’ standardized mean difference.

Variables that deviated from normality in either group were summarized as median and interquartile range and compared using the Mann–Whitney U test. Effect size was expressed as Cliff’s delta.

Categorical variables were summarized as n (%) and compared using Pearson’s chi-square test or Fisher’s exact test when expected counts were insufficient. Strength of association was expressed using Cramer’s V.

For the 4 × 4 cross-classification of ultrasonography score and study-defined MRI categories, Pearson’s chi-square statistic was calculated. Because of small expected cell counts, the *p*-value was estimated by Monte Carlo simulation with 10,000 replicates. Effect size was expressed as Cramer’s V.

Exact category agreement and agreement within ±1 category were summarized descriptively. Ordinal correspondence was assessed using linear- and quadratic-weighted Cohen’s kappa. Percentile bootstrap 95% confidence intervals were obtained from 2000 resamples.

Sensitivity, specificity, positive predictive value, negative predictive value, and overall accuracy were calculated from 2 × 2 tables. Wilson 95% confidence intervals were used for proportions. Positive and a negative likelihood ratios and diagnostic odds ratios were presented with 95% confidence intervals calculated on the logarithmic scale.

Discrimination of the ordinal ultrasonography score for the primary MRI outcome was evaluated using receiver operating characteristic analysis. The area under the curve and its 95% confidence interval were calculated using the DeLong method. A separate hypothesis test comparing the area under the curve with 0.5 was not performed.

Associations between the ordinal ultrasonography score and the continuous MRI-derived pancreatic fat index were evaluated using Spearman’s rank correlation and Kendall’s tau-b.

The MRI-derived pancreatic fat index was compared across ultrasonography score categories using the Kruskal–Wallis test. Effect size was expressed as epsilon-squared. When the global test was significant, pairwise Wilcoxon rank-sum tests with Holm correction were performed.

All tests were two-sided, and *p* < 0.05 was considered statistically significant. The random-number seed for Monte Carlo and bootstrap procedures was set to 20260726. Technical visualization yield and non-visualization rate were reported with Wilson 95% confidence intervals. Because MRI reference status was unavailable for the 20 technically inadequate examinations, exact full-cohort sensitivity and specificity were not estimable. Exploratory partial-identification bounds counted each non-diagnostic examination as an incorrect classification while allowing the number meeting the MRI endpoint to range from 0 to 20. An illustrative decision analysis used the observed confusion matrix and assumed that technically inadequate examinations would proceed directly to MRI; it was not a cost-effectiveness analysis.

### 2.10. Ethical Considerations

The study was conducted in accordance with the Declaration of Helsinki and was approved by the Local Bioethics Commission of Astana Medical University (Decision No. 3, Meeting No. 2, 29 January 2025).

Written informed consent for participation and use of de-identified results in scientific publications was obtained before enrollment. Data were de-identified before statistical analysis. This observational diagnostic accuracy study was not prospectively registered in a public study registry. Participants were not assigned to a health-related intervention, and the study was not registered as an interventional clinical trial.

## 3. Results

After nine clinical exclusions, 150 of 170 participants had technically adequate pancreatic ultrasonography, corresponding to a technical visualization yield of 88.2% (95% CI, 82.5–92.3%); 20 of 170 examinations (11.8%; 95% CI, 7.7–17.5%) were technically inadequate. The paired accuracy analysis therefore included 150 participants with evaluable ultrasonography and MRI. Of these, 68 (45.3%) had an MRI-reported pancreatic fat index < 5%, whereas 82 (54.7%) met the study-defined endpoint ≥ 5%.

Participants with MRI-reported pancreatic fat ≥ 5% were older than those with values below 5%, with median ages of 51.5 years [interquartile range (IQR), 42.2–60.0] and 40.0 years [IQR, 32.0–52.2], respectively (*p* < 0.001; Cliff’s δ = 0.377). The MRI ≥ 5% group also had a higher median body mass index than the MRI < 5% group: 29.65 kg/m^2^ [IQR, 25.91–33.26] versus 25.04 kg/m^2^ [IQR, 23.10–28.72] (*p* < 0.001; Cliff’s δ = 0.447). Similarly, median waist circumference was greater among participants with MRI-reported pancreatic fat ≥ 5%: 88.5 cm [IQR, 80.0–98.5] versus 80.5 cm [IQR, 67.8–89.0] (*p* < 0.001; Cliff’s δ = 0.325).

BMI-defined obesity was observed in 40 of 82 participants (48.8%) with MRI-reported pancreatic fat ≥ 5%, compared with 13 of 68 participants (19.1%) in the MRI < 5% group (*p* < 0.001; Cramer’s V = 0.309). No statistically significant between-group differences were identified for male sex, arterial hypertension, diabetes mellitus, or current smoking (Table 1).

Continuous variables are presented as median [interquartile range] and were compared using the Mann–Whitney U test. Categorical variables are presented as n (%) and were compared using Pearson’s chi-square test.

The distribution of the ordinal ultrasonography score differed significantly across the four study-defined MRI categories (Table 2). Among participants with MRI-reported pancreatic fat < 5%, 53 of 68 (77.9%) had a US score of 0. In contrast, a US score of 3 was recorded in 22 of 45 participants (48.9%) in the 5–<10% MRI category, 24 of 28 participants (85.7%) in the 10–<20% category, and 7 of 9 participants (77.8%) in the ≥20% category.

The overall association between the four-category MRI classification and the ordinal US score was statistically significant (Pearson χ^2^ = 68.170, df = 9; Monte Carlo *p* < 0.001), with a Cramer’s V of 0.389. Exact agreement between the US score and the study-defined MRI category was 45.3%, whereas agreement within one category was 76.7%. The cross-classification of the ultrasonography score and study-defined MRI pancreatic fat categories is additionally presented graphically in Figure S1 in the Supplementary Materials.

The linear-weighted Cohen’s kappa was 0.387 (95% confidence interval [CI], 0.298–0.474), and the quadratic-weighted kappa was 0.504 (95% CI, 0.387–0.612). Thus, agreement increased when larger discrepancies between the two ordinal classifications were assigned greater weight. Nevertheless, the results demonstrate that ultrasonography did not completely reproduce the MRI-based categorization.

Cells show n (row %). Pearson χ^2^ = 68.170, df = 9, Monte Carlo *p* < 0.001; Cramer’s V = 0.389. Linear-weighted kappa = 0.387 (95% CI 0.298–0.474); quadratic-weighted kappa = 0.504 (95% CI 0.387–0.612). Exact agreement was 45.3%, and agreement within ±1 category was 76.7%.

Using the primary rule of US score ≥ 1 to identify the study-defined two-echo MRI pancreatic fat index ≥ 5%, 63 participants were classified as true positive, 19 as false negative, 15 as false positive, and 53 as true negative (Table 3). Sensitivity was 76.8% (95% CI, 66.6–84.6%), specificity was 77.9% (95% CI, 66.7–86.2%), positive predictive value was 80.8% (95% CI, 70.7–88.0%), negative predictive value was 73.6% (95% CI, 62.4–82.4%), and overall accuracy was 77.3% (95% CI, 70.0–83.3%). These estimates apply only to the 150 technically evaluable participants.

For the primary threshold, the positive likelihood ratio was 3.48 (95% CI, 2.19–5.53), the negative likelihood ratio was 0.30 (95% CI, 0.20–0.45), and the diagnostic odds ratio was 11.72 (95% CI, 5.43–25.28).

Increasing the US-positive threshold improved specificity but reduced sensitivity. At US score ≥ 2, sensitivity decreased to 67.1% (95% CI, 56.3–76.3%), whereas specificity increased to 89.7% (95% CI, 80.2–94.9%). At US score ≥ 3, sensitivity was 64.6% (95% CI, 53.8–74.1%) and specificity was 91.2% (95% CI, 82.1–95.9%). The corresponding positive predictive values were 88.7% and 89.8%, respectively. Therefore, the higher exploratory thresholds were associated with fewer false-positive findings but a greater number of false-negative results.

**Figure 2:**
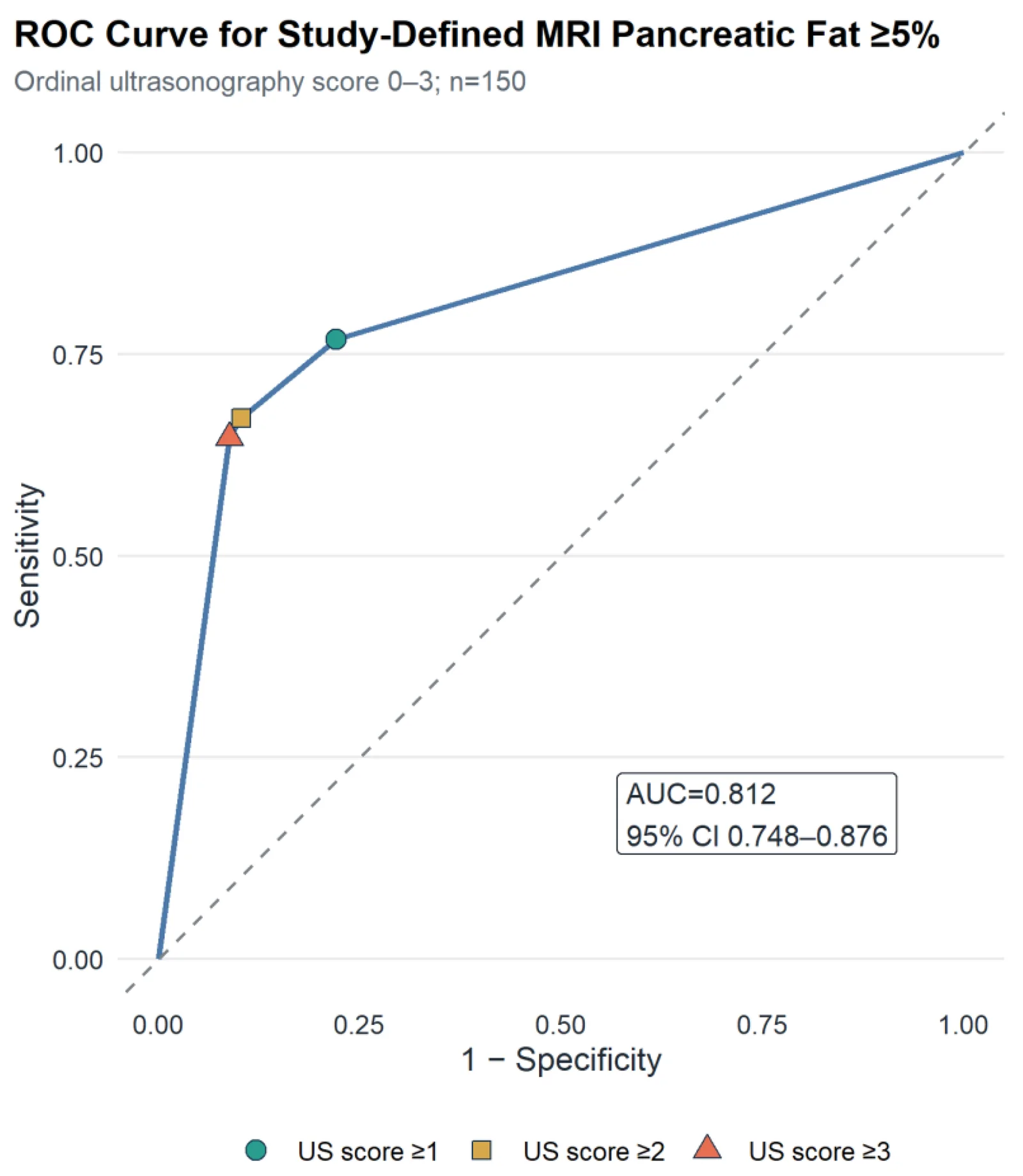
Receiver operating characteristic curve of the ordinal ultrasonography score for detecting study-defined MRI-reported pancreatic fat ≥ 5%. The area under the curve was 0.812 (95% CI 0.748–0.876). Marked points represent US-positive thresholds of ≥1, ≥2, and ≥3. The dashed diagonal line represents the line of no discrimination (AUC = 0.5).

For the study-defined MRI index ≥ 5%, the ordinal US score had an AUC of 0.812 (95% CI, 0.748–0.876) (Figure 2). The marked points on the ROC curve show the trade-off between sensitivity and specificity at US-positive thresholds ≥ 1, ≥2, and ≥3.

In the literature-informed sensitivity analysis using the MRI index > 6.2%, 52 participants were true positive, 15 false negative, 26 false positive, and 57 true negative. Sensitivity was 77.6% (95% CI, 66.3–85.9%), specificity was 68.7% (95% CI, 58.1–77.6%), accuracy was 72.7% (95% CI, 65.0–79.2%), and AUC was 0.773 (95% CI, 0.701–0.845). Thus, the overall finding persisted at >6.2%, although specificity was lower than at the study-defined 5% threshold. Additional sensitivity analyses are provided in Table S1, and sensitivity and specificity across ultrasonography score thresholds are illustrated in Figure S2 in the Supplementary Materials.

The ordinal US score yielded an AUC of 0.812 (95% CI 0.748–0.876) for MRI-reported pancreatic fat ≥ 5%. Proportion confidence intervals were calculated using the Wilson method.

In an illustrative pathway in which MRI would be performed only after a technically evaluable US score ≥ 1, 78 of 150 participants would proceed to MRI and 72 of 150 (48.0%) MRI examinations would be avoided; however, 19 of 82 (23.2%) participants meeting the MRI index ≥ 5% would be missed. If every technically inadequate US examination were instead referred directly to MRI, 98 of 170 participants (57.6%) would undergo MRI and 72 of 170 (42.4%) would avoid MRI. These calculations are descriptive and do not incorporate costs or clinical outcomes.

MRI-reported pancreatic fat values increased across the ordinal US score categories (Table 4). The median MRI-reported pancreatic fat value was 2.80% [IQR, 1.37–5.38%] among participants with a US score of 0, 4.95% [IQR, 3.30–6.90%] among those with a score of 1, 7.00% [IQR, 5.50–7.05%] among those with a score of 2, and 10.70% [IQR, 6.40–15.38%] among those with a score of 3.

The overall difference across the four US score groups was statistically significant (Kruskal–Wallis H = 50.241, *p* < 0.001), with an epsilon-squared effect size of 0.324. A positive monotonic association was also observed between the ordinal US score and continuous MRI-reported pancreatic fat, as demonstrated by Spearman’s correlation coefficient (ρ = 0.574, *p* < 0.001) and Kendall’s tau-b (τ-b = 0.468, *p* < 0.001).

After adjustment for multiple comparisons using the Holm method, participants with a US score of 3 had significantly higher MRI-reported pancreatic fat values than those with scores of 0 and 1 (both adjusted *p* < 0.001). The remaining pairwise comparisons were not statistically significant after adjustment. Comparisons involving US score 2 should be interpreted cautiously because this category included only three participants.

**Figure 3:**
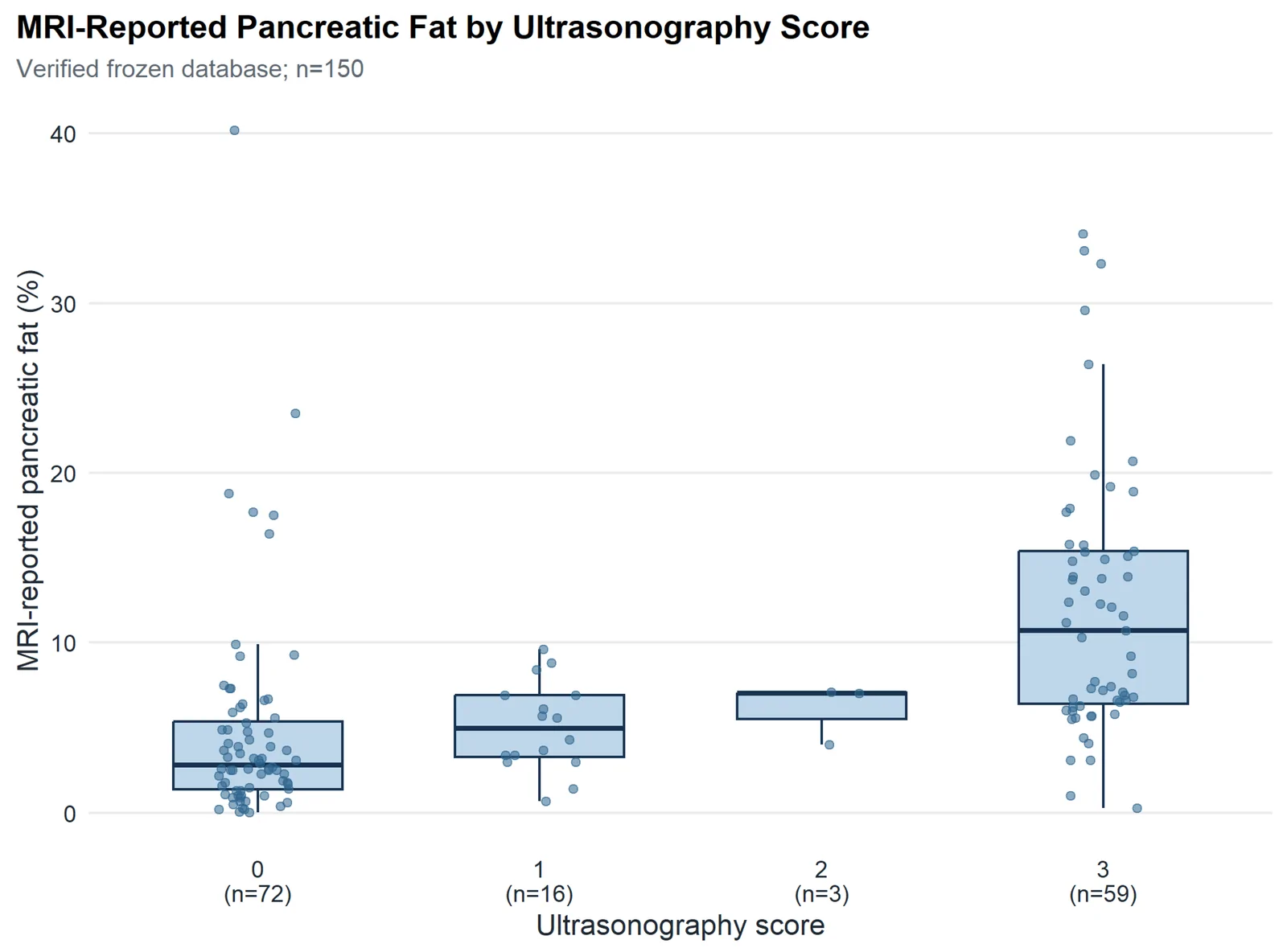
Distribution of MRI-reported pancreatic fat according to the ultrasonography score. Boxes represent the median and interquartile range, whiskers extend to 1.5 times the interquartile range, and jittered points represent individual participants. Only three participants had US score 2.

Although the median MRI-reported pancreatic fat value increased with the US score, considerable overlap between the score categories remained (Figure 3). These findings indicate that the ordinal US score reflects the overall direction and increasing burden of MRI-reported pancreatic fat but does not provide an interchangeable quantitative estimate of the MRI measurement.

Kruskal–Wallis H = 50.241, *p* < 0.001; ε^2^ = 0.324. Spearman ρ = 0.574, *p* < 0.001; Kendall τ-b = 0.468, *p* < 0.001. After Holm correction, US score 3 differed significantly from scores 0 and 1; comparisons involving score 2 were inconclusive because only three participants had score 2.

## 4. Discussion

In this prospective paired study, the ordinal transabdominal ultrasonography score had an AUC of 0.812 for identifying a study-defined semiquantitative two-echo MRI pancreatic fat index ≥ 5%; the primary threshold ≥ 1 yielded sensitivity of 76.8% and specificity of 77.9% among 150 technically evaluable participants. Exact agreement across four operational categories was 45.3%, and weighted kappa indicated only moderate ordinal correspondence. The central finding is therefore agreement and discrimination relative to this specific study-defined MRI index, not validation of ultrasonography for diagnosing a disease entity or reproducing quantitative MRI grading.

The primary US threshold ≥ 1 was anchored to the original binary clinical interpretation of diffuse pancreatic changes rather than selected from the observed ROC curve. In contrast, the MRI index ≥ 5% was a pragmatic operational boundary for this study and not an accepted clinical cutoff. The sensitivity analysis at >6.2% produced similar sensitivity (77.6%) but lower specificity (68.7%), demonstrating that the reported performance depends on the MRI definition. The 0–3 US score was retrospectively standardized and should be considered hypothesis-generating until reproducibility is established.

The findings are consistent with previous reports associating increased pancreatic echogenicity with pancreatic fat accumulation, obesity, abdominal adiposity, and other metabolic abnormalities [7,8,9,10,11,15,20,21,22]. Earlier studies frequently classified the pancreas simply as normal or hyperechoic, while the ultrasonographic signs, comparison organs, and confirmatory imaging methods differed substantially [12,15,19,20,21,22]. Lin et al. reported a kappa of 0.760 for an improved ultrasonography method compared with MRI mDIXON-Quant in 46 participants, whereas agreement in the present four-category analysis was more moderate; differences in scale construction, MRI technique, thresholds, sample size, and category structure preclude direct performance ranking [34]. The present study extends this literature by applying paired ultrasonography and MRI within one day, reporting a full set of diagnostic accuracy measures, and examining both binary performance and ordinal correspondence. This broader literature also shows why comparisons among studies should remain cautious: reported prevalence, echogenicity grades, and metabolic associations depend on the population, ultrasound technique, and reference method used [5,13,24,25].

The reference measure also constrains interpretation of the accuracy estimates. The two-echo signal-intensity calculation provided a consistent comparator but did not correct T1 weighting, T2* decay, noise bias, the multi-peak fat spectrum, or B0/B1 inhomogeneity and was not standardized MRI-PDFF. These physical confounders and manual ROI placement may have shifted individual values across the 5% boundary. Such reference-measure misclassification can change sensitivity, specificity, and AUC in either direction. Accordingly, the reported metrics represent performance against this study-defined semiquantitative reference measure rather than accuracy against histology, true tissue fat concentration, or standardized MRI-PDFF. Contemporary MRI methodology studies support this caution, because pancreatic fat estimates are affected by ROI strategy, sequence harmonization, reconstruction method, and correction of low-fat signal confounders [30,31,32,33].

The median MRI-derived pancreatic fat index increased from 2.80% at an ultrasonography score of 0 to 10.70% at a score of 3. The positive monotonic association was supported by Spearman’s ρ of 0.574 and Kendall’s tau-b of 0.468. Biologically, increasing fat accumulation may contribute to greater echogenicity, heterogeneous echotexture, acoustic attenuation, and reduced visualization of pancreatic margins. However, the association remained moderate and the distributions overlapped considerably. Ultrasonographic appearance is likely to reflect a combination of fat, body habitus, pancreatic depth, intestinal gas, equipment settings, heterogeneous fat distribution, and possible fibrosis [12,27,28,29]. This explains why the score tracked the general direction of change but did not provide a direct estimate of MRI-derived fat.

The primary likelihood ratios and illustrative decision analysis support only a cautious referral concept. A positive likelihood ratio of 3.48 and negative likelihood ratio of 0.30 do not establish or exclude the study-defined MRI endpoint. Restricting MRI to US-positive participants would avoid 48.0% of MRI examinations among the technically evaluable cohort but would miss 23.2% of participants meeting the MRI index ≥ 5%. In a pathway where technically inadequate US examinations proceed directly to MRI, the MRI avoidance fraction would be 42.4% among all 170 participants remaining after clinical exclusions. These figures do not demonstrate clinical utility or cost-effectiveness.

Raising the positive ultrasonography threshold to at least 2 or at least 3 increased specificity to 89.7% and 91.2%, respectively, but decreased sensitivity to 67.1% and 64.6%. Higher scores may therefore identify a subgroup with a greater probability of elevated pancreatic fat and could support prioritization for MRI. However, these thresholds are unsuitable for exclusion because they increase false-negative findings. Their interpretation is additionally constrained by the very small number of participants with a score of 2.

The four-category analysis demonstrated a statistically significant association with a moderate effect size, but the categories should not be considered interchangeable. Exact agreement was 45.3%, agreement within one category was 76.7%, and quadratic-weighted kappa was 0.504. These statistics describe correspondence between two classification systems, not agreement between readers. Moreover, the ultrasonography scale was based on qualitative imaging signs, whereas the MRI categories were operational intervals of a semiquantitative signal-intensity index. The moderate kappa therefore indicates ordered association, not equivalence or validated severity staging.

Participants with the primary MRI endpoint were older and had higher BMI and waist circumference, and BMI-defined obesity was more common. These observations align with the established relationship between pancreatic fat, age, general adiposity, abdominal adiposity, and metabolic dysfunction [2,3,6,7,8,9,10,11,22]. The comparisons were unadjusted and were not designed to identify independent predictors. Age, BMI, waist circumference, and obesity should therefore be described as associated factors rather than independently confirmed determinants. Similar associations with obesity, visceral adiposity, and future dysglycemia have been reported in recent MRI cohorts and meta-analytic literature [13,14,17,35].

From a practical perspective, transabdominal ultrasonography may provide an initial qualitative assessment in adults aged 30–60 years with metabolic risk when pancreatic visualization is technically adequate. However, technical non-visualization occurred in 11.8%, negative examinations missed 23.2% of participants meeting the study-defined MRI endpoint, and category correspondence was incomplete. The score should therefore not be used as a direct substitute for MRI grading, as an independent quantitative measurement of pancreatic fat, or as a validated diagnostic test for fatty pancreas in unselected populations.

The sensitivity analysis that counted every technically non-diagnostic examination as an incorrect classification produced partial-identification bounds because MRI status was unknown for these 20 participants. Depending on their unobserved MRI status, sensitivity could range from 61.8% to 76.8% and specificity from 60.2% to 77.9%; accuracy would be 68.2% under this conservative assumption. These bounds are not substitute estimates but show how excluding technical failures can make complete-case performance appear more favorable.

### 4.1. Strengths

Major strengths include prospective data collection, paired ultrasonography and MRI within one day, and inclusion of 150 participants with complete results for both methods. All included participants underwent the reference measure irrespective of the index-test result, reducing the risk of partial verification bias. MRI findings were unavailable during the original ultrasonography interpretation and subsequent score standardization, while MRI readers did not receive the ultrasonography results.

The analysis was comprehensive. In addition to sensitivity and specificity, it reported predictive values, accuracy, likelihood ratios, diagnostic odds ratios, and 95% confidence intervals. The ordinal score was further examined using receiver operating characteristic analysis, rank correlations, comparison of the continuous MRI-derived index across score categories, a 4 × 4 cross-classification, and weighted kappa. This allowed the study to distinguish diagnostic discrimination from ordinal correspondence.

Use of the same MRI scanner, sequence, and calculation procedure for all participants improved internal consistency. Complete data and reporting according to STARD 2015 also enhanced transparency regarding participant flow, test procedures, blinding, thresholds, and diagnostic outcomes.

### 4.2. Limitations

This study has several limitations. First, it involved a selected cohort of adults aged 30–60 years with metabolic abnormalities or risk factors and technically adequate ultrasonography. Although ultrasonography and MRI were performed at two institutions, this was not independent multicenter validation. The estimates should not be generalized to unselected populations, other age groups, individuals without metabolic risk, or settings with different rates of technical non-visualization. Predictive values will also vary with endpoint prevalence.

Second, recruitment combined referred patients, preventive-screening participants, and specifically invited individuals. The sample was therefore neither strictly consecutive nor random and may be affected by selection and spectrum bias.

Third, the paired analysis included only participants with technically adequate results. Technical visualization was successful in 150 of 170 participants (88.2%), and excluding the remaining 20 may have selected a more readily examinable population and overestimated routine-practice performance. Their individual BMI and other characteristics and MRI reference status were not available in the finalized analytical dataset, preventing direct comparison with included participants and an exact full-cohort accuracy analysis. The reported scenario bounds should be viewed as exploratory rather than as observed diagnostic estimates.

Fourth, the ordinal 0–3 ultrasonography score was retrospectively standardized from original reports and stored images and had not been previously validated. The two assessors reviewed cases jointly and assigned consensus scores; independent initial or repeated ratings were not obtained. Consequently, inter-reader and intra-reader reproducibility could not be estimated, and the ordinal analyses remain exploratory.

Fifth, the reference measure was a semiquantitative two-echo chemical-shift MRI index without corrections for T1 weighting, T2* decay, noise bias, the multi-peak fat spectrum, or B0/B1 inhomogeneity. It was not standardized MRI-PDFF. Independent duplicate ROI placement was not performed, so MRI measurement reproducibility was not quantified. Reference-measure error may have caused threshold misclassification and affected sensitivity, specificity, and AUC in an unknown direction. Thus, the reported values characterize agreement with the study-defined MRI index, not accuracy relative to histology or standardized MRI-PDFF.

Sixth, alcohol exposure was based on clinical interview, self-report, and available medical records. A validated quantitative instrument was not used, and the exclusion thresholds were relatively high. Residual misclassification of alcohol exposure cannot be excluded and should be considered when interpreting the nonalcoholic classification.

Seventh, some categories were sparse. Only three participants had an ultrasonography score of 2, and only nine met the MRI index ≥ 20%. The sparse occupancy of score 2 may reflect the retrospective scale construction and precludes firm conclusions that all four US categories distinguish stable severity levels. All multicategory, ordinal, and ≥20% analyses should therefore be interpreted as exploratory or descriptive.

Finally, no a priori sample-size calculation was performed specifically for diagnostic accuracy. The retrospective Buderer calculation describes only the precision achieved for the primary binary analysis and does not justify the sparse ordinal severity analyses. No external validation was available.

### 4.3. Future Research

Future studies should prospectively recruit independent multicenter cohorts, prespecify the ultrasonography scale, standardize equipment settings and image-quality criteria, and record technically inadequate examinations as clinically meaningful test outcomes. Multiple readers blinded to MRI should independently assess images, with weighted kappa used to quantify inter-reader and intra-reader reproducibility.

MRI assessment should preferably use standardized multi-echo sequences with correction of major physical confounders and a documented ROI protocol. Independent repeat measurements would allow inter-reader reproducibility to be quantified using the intraclass correlation coefficient. External calibration against histology, spectroscopy, or standardized MRI-PDFF should be considered when clinically and ethically feasible. Recent harmonization and validation studies support the use of standardized, confounder-corrected chemical-shift-encoded MRI with reproducible whole-pancreas or prespecified regional sampling strategies in future work [30,31,33].

Combining ultrasonographic signs with age, BMI, waist circumference, and laboratory markers of metabolic risk may improve selection for MRI. Any multivariable model should be developed with appropriate control of overfitting and undergo internal and external validation. Additional priorities include defining clinically meaningful MRI thresholds, evaluating longitudinal change, and determining whether pancreatic fat adds prognostic information beyond general and visceral adiposity.

## 5. Conclusions

Among 150 adults aged 30–60 years with metabolic risk factors and technically adequate ultrasonography, the ordinal score had an AUC of 0.812 (95% CI, 0.748–0.876) for detecting the study-defined semiquantitative two-echo MRI pancreatic fat index ≥ 5%. A US score ≥ 1 yielded sensitivity of 76.8% (95% CI, 66.6–84.6%), specificity of 77.9% (95% CI, 66.7–86.2%), and accuracy of 77.3% (95% CI, 70.0–83.3%). Pancreatic visualization was technically adequate in 88.2% of participants remaining after clinical exclusions.

Higher ultrasonography scores were associated with higher MRI-derived index values, but exact agreement across the four operational categories was only 45.3% and quadratic-weighted kappa was 0.504. These findings indicate moderate ordinal correspondence, not equivalence or validated severity staging.

The reported performance applies only to the study-defined two-echo MRI index in this selected, technically evaluable cohort. It should not be interpreted as diagnostic accuracy for histologically confirmed pancreatic steatosis, a validated clinical disease definition, or standardized MRI-PDFF-defined disease. The exploratory 0–3 score is not a quantitative substitute for MRI and requires prospective reproducibility assessment and external validation before clinical use.

## Figures and Tables

**Table 1:** Participant Characteristics by the Primary MRI Endpoint.

Characteristic	Overall (*n* = 150)	MRI < 5% (*n* = 68)	MRI ≥ 5% (*n* = 82)	*p* Value	Effect Size
Age, years	47.0 [36.0–58.0]	40.0 [32.0–52.2]	51.5 [42.2–60.0]	<0.001	Cliff’s δ = 0.377
Body mass index, kg/m^2^	27.75 [24.28–31.56]	25.04 [23.10–28.72]	29.65 [25.91–33.26]	<0.001	Cliff’s δ = 0.447
Waist circumference, cm	85.0 [73.0–94.5]	80.5 [67.8–89.0]	88.5 [80.0–98.5]	<0.001	Cliff’s δ = 0.325
Male sex	50 (33.3%)	19 (27.9%)	31 (37.8%)	0.202	Cramer’s V = 0.104
Arterial hypertension	32 (21.3%)	14 (20.6%)	18 (22.0%)	0.839	Cramer’s V = 0.017
Diabetes mellitus	14 (9.3%)	3 (4.4%)	11 (13.4%)	0.059	Cramer’s V = 0.154
Current smoking	30 (20.0%)	13 (19.1%)	17 (20.7%)	0.806	Cramer’s V = 0.020
BMI-defined obesity	53 (35.3%)	13 (19.1%)	40 (48.8%)	<0.001	Cramer’s V = 0.309

**Table 2:** Ultrasonography Score by Study-Defined MRI Category.

MRI Category	US Score 0	US Score 1	US Score 2	US Score 3	Total
<5%	53 (77.9%)	8 (11.8%)	1 (1.5%)	6 (8.8%)	68
5–<10%	13 (28.9%)	8 (17.8%)	2 (4.4%)	22 (48.9%)	45
10–<20%	4 (14.3%)	0 (0.0%)	0 (0.0%)	24 (85.7%)	28
≥20%	2 (22.2%)	0 (0.0%)	0 (0.0%)	7 (77.8%)	9

**Table 3:** Diagnostic Performance for MRI-Reported Pancreatic Fat ≥ 5%.

US-Positive Rule	Role	TP/FN/FP/TN	Sensitivity, %	Specificity, %	PPV, %	NPV, %	Accuracy, %	LR+	LR−	DOR
US score ≥1	Primary	63/19/15/53	76.8 (66.6–84.6)	77.9 (66.7–86.2)	80.8 (70.7–88.0)	73.6 (62.4–82.4)	77.3 (70.0–83.3)	3.48 (2.19–5.53)	0.30 (0.20–0.45)	11.72 (5.43–25.28)
US score ≥2	Exploratory	55/27/7/61	67.1 (56.3–76.3)	89.7 (80.2–94.9)	88.7 (78.5–94.4)	69.3 (59.0–78.0)	77.3 (70.0–83.3)	6.52 (3.18–13.36)	0.37 (0.27–0.51)	17.75 (7.16–44.00)
US score ≥3	Exploratory	53/29/6/62	64.6 (53.8–74.1)	91.2 (82.1–95.9)	89.8 (79.5–95.3)	68.1 (58.0–76.8)	76.7 (69.3–82.7)	7.33 (3.36–15.99)	0.39 (0.29–0.52)	18.89 (7.29–48.95)

**Table 4:** MRI-Reported Pancreatic Fat According to the Ultrasonography Score.

US Score	n	MRI Fat, Median [IQR], %	Range, %
0	72	2.80 [1.37–5.38]	0.02–40.2
1	16	4.95 [3.30–6.90]	0.7–9.6
2	3	7.00 [5.50–7.05]	4.0–7.1
3	59	10.70 [6.40–15.38]	0.3–34.1

## Data Availability

The de-identified data supporting the findings of this study are available from the corresponding author upon reasonable request, subject to ethical and institutional restrictions.

## Supplementary Materials

The following supporting information can be downloaded at: https://www.mdpi.com/article/10.3390/diagnostics16182959/s1, Table S1: MRI Endpoint Sensitivity Analyses; Figure S1: Cross-classification of the ultrasonography score and study-defined MRI pancreatic fat categories. Cells show patient counts and row percentages; Figure S2: Sensitivity and specificity across ultrasonography score thresholds for detecting a study-defined MRI-derived pancreatic fat index ≥5%. Error bars represent 95% confidence intervals; Checklist S1: STARD 2015 checklist.
