# Supplementary material for: Diagnostic Performance of Transabdominal Ultrasonography for Pancreatic Fat in Adults with Metabolic Risk Factors for NAFPD: Comparison with a Study-Defined Two-Echo MRI Index
Source: Diagnostics (Basel). 2026 Sep 13;16(18):2959. doi: 10.3390/diagnostics16182959 (PMC13605573; doi:10.3390/diagnostics16182959)
Supplement: Supplementary file 1 [file diagnostics-16-02959-s001.zip › Checklist S1.pdf]

Checklist S1

STARD 2015 Checklist

Diagnostic Performance of Transabdominal Ultrasonography for Pancreatic Fat in Adults with Metabolic Risk Factors for NAFPD: Comparison with a Study-Defined Two-Echo MRI Index

Section & topic	No.	STARD 2015 item	Location in manuscript
**TITLE OR ABSTRACT**	1	Identification as a study of diagnostic accuracy using at least one measure of accuracy (such as sensitivity, specificity, predictive values, or AUC)	Title; Abstract
**ABSTRACT**	2	Structured summary of study design, methods, results, and conclusions (for specific guidance, see STARD for Abstracts)	Abstract
**INTRODUCTION**	3	Scientific and clinical background, including the intended use and clinical role of the index test	Section 1, Introduction
**INTRODUCTION**	4	Study objectives and hypotheses	Section 1, final paragraph
**METHODS – Study design**	5	Whether data collection was planned before the index test and reference standard were performed (prospective study) or after (retrospective study)	Sections 2.1 and 2.4
**METHODS – Participants**	6	Eligibility criteria	Section 2.2
**METHODS – Participants**	7	On what basis potentially eligible participants were identified (such as symptoms, results from previous tests, inclusion in a registry)	Sections 2.1 and 2.2
**METHODS – Participants**	8	Where and when potentially eligible participants were identified (setting, location, and dates)	Section 2.1
**METHODS – Participants**	9	Whether participants formed a consecutive, random, or convenience series	Section 2.1
**METHODS – Test methods**	10a	Index test, in sufficient detail to allow replication	Section 2.4
**METHODS – Test methods**	10b	Reference standard, in sufficient detail to allow replication	Section 2.5
**METHODS – Test methods**	11	Rationale for choosing the reference standard (if alternatives exist)	Section 1; Section 2.5
**METHODS – Test methods**	12a	Definition of and rationale for test positivity cut-offs or result categories of the index test, distinguishing pre-specified from exploratory	Sections 2.4 and 2.7

Section & topic	No.	STARD 2015 item	Location in manuscript
**METHODS – Test methods**	12b	Definition of and rationale for test positivity cut-offs or result categories of the reference standard, distinguishing pre-specified from exploratory	Sections 2.5 and 2.7; Table S1
**METHODS – Test methods**	13a	Whether clinical information and reference standard results were available to the performers/readers of the index test	Section 2.4
**METHODS – Test methods**	13b	Whether clinical information and index test results were available to the assessors of the reference standard	Section 2.5
**METHODS – Analysis**	14	Methods for estimating or comparing measures of diagnostic accuracy	Sections 2.7 and 2.9
**METHODS – Analysis**	15	How indeterminate index test or reference standard results were handled	Sections 2.2, 2.4, and 2.6; Figure 1
**METHODS – Analysis**	16	How missing data on the index test and reference standard were handled	Sections 2.6 and 2.8
**METHODS – Analysis**	17	Any analyses of variability in diagnostic accuracy, distinguishing pre-specified from exploratory	No prespecified reader- or participant-subgroup variability analysis; exploratory MRI-threshold sensitivity analyses: Section 2.7; Table S1; Figure S2
**METHODS – Analysis**	18	Intended sample size and how it was determined	Section 2.8
**RESULTS – Participants**	19	Flow of participants, using a diagram	Section 2.2; Figure 1
**RESULTS – Participants**	20	Baseline demographic and clinical characteristics of participants	Results; Table 1
**RESULTS – Participants**	21a	Distribution of severity of disease in those with the target condition	Results; Tables 2 and 4; Figure 3
**RESULTS – Participants**	21b	Distribution of alternative diagnoses in those without the target condition	Not applicable; major alternative pancreatic and hepatic conditions were exclusion criteria (Section 2.2)
**RESULTS – Participants**	22	Time interval and any clinical interventions between index test and reference standard	Section 2.6

Section & topic	No.	STARD 2015 item	Location in manuscript
**RESULTS – Test results**	23	Cross tabulation of the index test results (or their distribution) by the results of the reference standard	Results; Tables 2 and 3; Figure S1
**RESULTS – Test results**	24	Estimates of diagnostic accuracy and their precision (such as 95% confidence intervals)	Results; Table 3; Figure 2; Table S1
**RESULTS – Test results**	25	Any adverse events from performing the index test or the reference standard	Section 2.6
**DISCUSSION**	26	Study limitations, including sources of potential bias, statistical uncertainty, and generalisability	Section 4.2
**DISCUSSION**	27	Implications for practice, including the intended use and clinical role of the index test	Section 4; Section 5
**OTHER INFORMATION**	28	Registration number and name of registry	Not prospectively registered in a public study registry; not registered as an interventional clinical trial; Institutional Review Board Statement.
**OTHER INFORMATION**	29	Where the full study protocol can be accessed	No publicly accessible study protocol is available; the study procedures are described in detail in Section 2, Materials and Methods.
**OTHER INFORMATION**	30	Sources of funding and other support; role of funders	Funding statement
