# Supplementary material for: Diagnostic Performance of Transabdominal Ultrasonography for Pancreatic Fat in Adults with Metabolic Risk Factors for NAFPD: Comparison with a Study-Defined Two-Echo MRI Index
Source: Diagnostics (Basel). 2026 Sep 13;16(18):2959. doi: 10.3390/diagnostics16182959 (PMC13605573; doi:10.3390/diagnostics16182959)
Supplement: Supplementary file 1 [file diagnostics-16-02959-s001.zip › Supplementary Materials.pdf]

Supplementary Materials

Diagnostic Performance of Transabdominal Ultrasonography for Pancreatic Fat in Adults with Metabolic Risk Factors for NAFPD: Comparison with a Study-Defined Two-Echo MRI Index

Anel Zhangereyeva, Venera Rakhmetova, Nurlan Baisynov and Aitore Zhangereyev

Table S1. MRI Endpoint Sensitivity Analyses

MRI endpoint	Positive/negative, n	TP/FN/FP/TN	Sensitivity, % (95% CI)	Specificity, % (95% CI)	Accuracy, % (95% CI)	Analysis status
MRI ≥5%	82/68	63/19/15/53	76.8 (66.6–84.6)	77.9 (66.7–86.2)	77.3 (70.0–83.3)	Primary analysis
MRI >6.2%	67/83	52/15/26/57	77.6 (66.3–85.9)	68.7 (58.1–77.6)	72.7 (65.0–79.2)	Sensitivity analysis
MRI ≥10%	37/113	31/6/47/66	83.8 (68.9–92.3)	58.4 (49.2–67.1)	64.7 (56.7–71.9)	Secondary analysis
MRI ≥20%	9/141	7/2/71/70	77.8 (45.3–93.7)	49.6 (41.5–57.8)	51.3 (43.4–59.2)	Descriptive analysis; sparse data

Note: US score ≥1 was considered a positive ultrasonographic result. The MRI ≥5% threshold represented the primary operational endpoint. The literature-informed threshold of >6.2% was evaluated in a sensitivity analysis. MRI thresholds ≥10% and ≥20% were evaluated as secondary and descriptive thresholds, respectively. The ≥20% analysis should be interpreted cautiously because only nine participants met this endpoint. Confidence intervals were calculated using the Wilson method. TP, true positive; FN, false negative; FP, false positive; TN, true negative; CI, confidence interval; MRI, magnetic resonance imaging; US, ultrasonography.

Figure S1

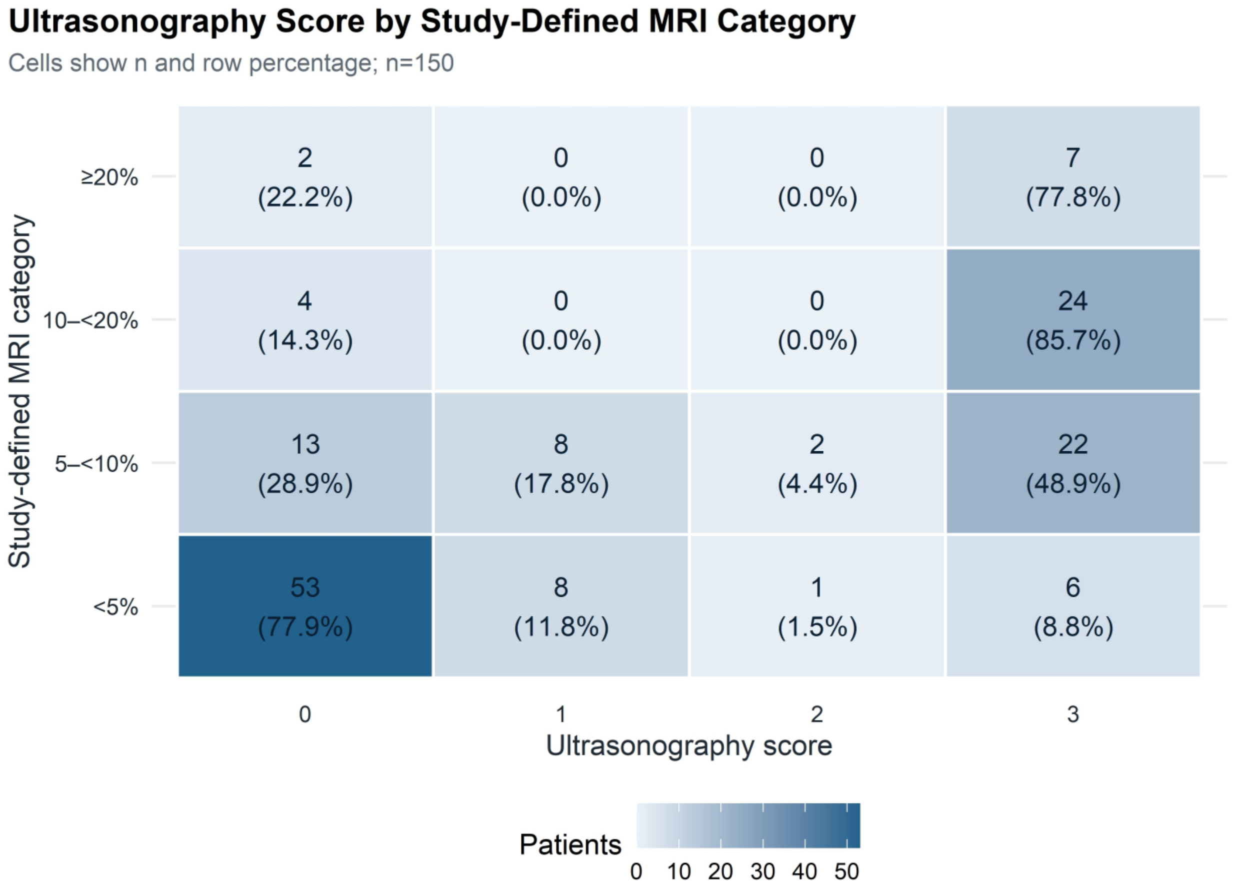

Figure S1. Cross-classification of the ultrasonography score and study-defined MRI pancreatic fat categories. Cells show patient counts and row percentages.

Figure S2

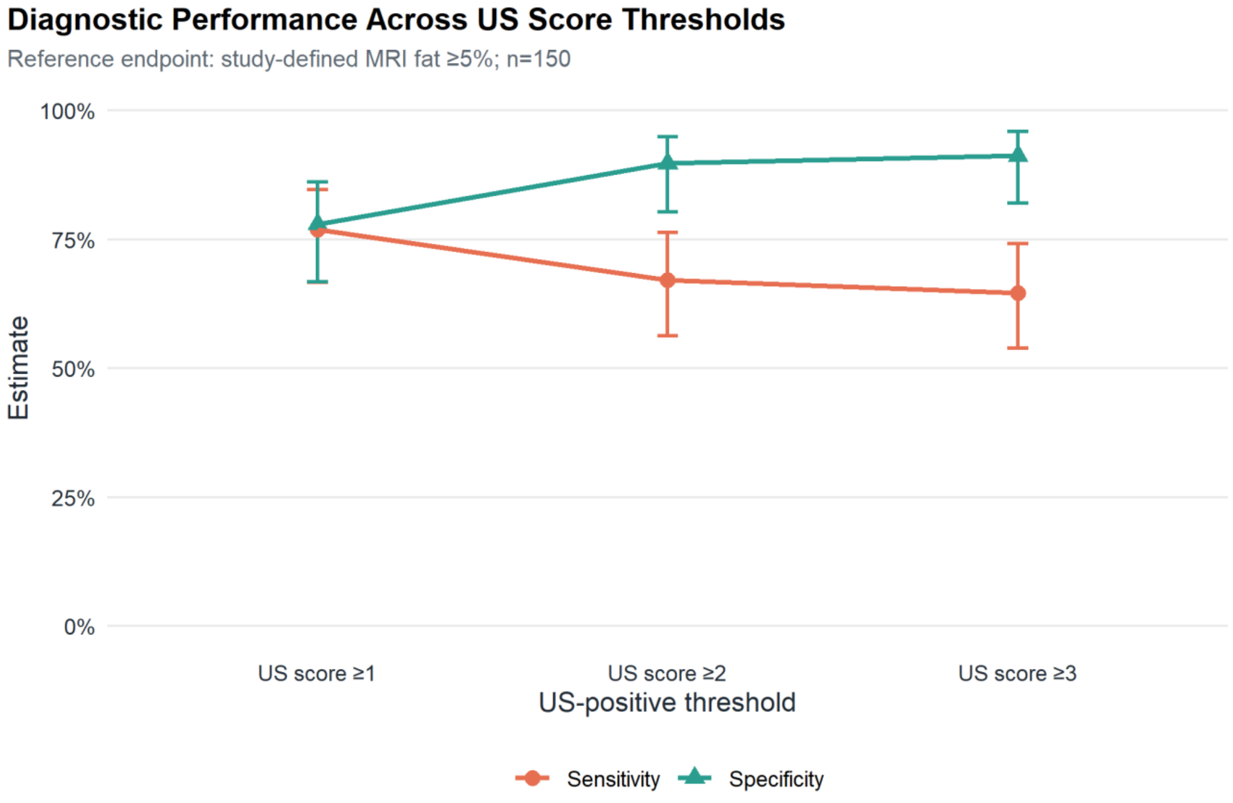

Figure S2. Sensitivity and specificity across ultrasonography score thresholds for detecting a study-defined MRI-derived pancreatic fat index ≥5%. Error bars represent 95% confidence intervals.
